# Minoxidil induced hypertrichosis in children

**DOI:** 10.11604/pamj.2014.18.8.3637

**Published:** 2014-05-02

**Authors:** Najwa Guerouaz, Ait Ourhroui Mohamed

**Affiliations:** 1 University hospital Ibn Sina, Department of Dermatology, Morocco

**Keywords:** Minoxidil, hypertrichosis, side-effects

## Image in medicine

Minoxidil is a strong arterial vasodilator used in the treatment of hypertension. In dermatology, topic minoxidil is widely used to treat hair fall. Hypertrichosis may occur if this form is misused. A 5 year-old girl developed patchy alopecia areata of the scalp. She was treated by minoxidil lotion 2% 10 times a day without medical prescription. Regrow of hair was rapidly obtained. However, the parents complained of extensive hypertrichosis covering the face (A, B) and the back (C). Physical examination revealed no hypotension and hirsutism evaluated at 20/36 on the Ferriman and Gallwey scale. Serum levels of 17 hydroxyprogesterone, 17alfa progesterone, testosterone and cotisol were within normal ranges excluding hormonal disorder. Discontinuation of minoxidil was followed by progressive regression of hypertrichosis. Systemic effects are rarely reported with minoxidil lotions. Indeed, transcutaneous absorption is insignificant ranging from 0,3 to 4,5%. The main systemic side effects include hypotension, tachycardia and ECG changes. Hypertrichosis has been reported more frequently in females than in males. This side effect is dose-related and is manly localized in the face. Discontinuation of treatment makes this trouble reversible. However medical supervision must be considered for a long time because of the product's very long action. Minoxidil must be delivered on medical prescription to ensure the maximum security use especially in children and women.

**Figure 1:**
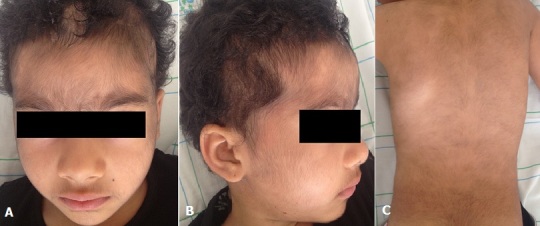
A) Hypertrichosis of the face; B) Hypertrichosis of the face; C) Hypertrichosis of the back

